# Nutrient profiles, functional compositions, and antioxidant activities of seven types of grain fermented with *Sanghuangporus sanghuang* fungus

**DOI:** 10.1007/s13197-020-04868-7

**Published:** 2020-11-11

**Authors:** Tingting Song, Zuofa Zhang, Qunli Jin, Weilin Feng, Yingyue Shen, Lijun Fan, Weiming Cai

**Affiliations:** Institute of Horticulture, Zhejiang Academy of Agricultural Sciences, Hangzhou, 310021 Zhejiang People’s Republic of China

**Keywords:** *Sanghuangporus sanghuang*, Solid-state fermentation (SSF), Functional compositions, Antioxidant capacities

## Abstract

*Sanghuangporus sanghuang* (SS) is a rare medicinal polypore fungus that grows solely on *Morus* trees. In this study, seven grains (oats, barley, millet, rice, buckwheat, corn, and coix seed) were used as solid substrates for SS fermentation and characterized in their nutrition, functional composition, and antioxidant activities. After fermentation, the nutrient compositions of crude protein (*F*_1,41_ = 111.1, *P* < 0.01), soluble protein (*F*_1,41_ = 595.7, *P* < 0.01), soluble sugar (*F*_1,41_ = 51.4, *P* < 0.01) and ash (*F*_1,41_ = 227.3, *P* < 0.01) increased significantly. Oats were one of the best grains for SS fermentation, SS-Oat produced 6.23 mg QE/g polyphenols, 21.8 mg rutin/g flavonoids, and 2.3% triterpene. In addition, the antioxidant capacities of the seven grains all increased. Principal component analysis analysis shows that the antioxidant properties of the grains were similar after SS fermentation. The changes of antioxidant activity due to SS fermentation were corrected with corresponding grain and remarked as *Δ*T-AOC/ABTS^+^/DPPH/DNAp, that was correlated to part of changes in polyphenol, carotenoid, triterpenoids, and flavonoid contents. In summary, oats have the greatest potential for use as a fermentation substrate for health food development.

## Introduction

Edible mushrooms are rich in nutrients and have therapeutic value. There is one of the most popular medicinal polypore groups mushroom named ‘sanghuang’ in China herbal markets. Taxonomically, the ‘sanghuang’ groups belongs to phylum Basidiomycetes, class Hymenomycetes, order Polyporales, family Polyporaceae, and genus *Sanghuangporus* (Zhou et al. 2016). *Sanghuangporus sanghuang* (SS) is one of most important species in the ‘sanghuang’ group, which only grow in *Morus*. It is rare in Asia and famous for its medicinal properties (Wu et al. 2012).

As a macrofungus, SS contains various bioactive compounds, including polyphenols, triterpenoids, and steroids, which have a wide range of biological activities. They can have anti-inflammatory and anti-angiogenetic effects, repair cell damage, enhance immune function, and inhibit tumor growth and metastasis in vitro and in vivo (Zhu et al. 2007; Silva et al. 2008; Ohno et al. 2007). Furthermore, these secondary metabolites have the ability to scavenge free radicals and are recognized as excellent antioxidants (Ejelonu et al. 2013).

Free radicals are one of the most important factors in the pathogenesis of various diseases that disturb normal human functioning and damage cellular organic molecules (e.g. lipids, proteins, and DNA). So, the search for new products with antioxidant properties is a very active domain of research. In recent decades, much research has shown that macrofungi possess the ability to clear free radicals (Ejelonu et al. 2013), in which phenolics are one of major secondary metabolites and have antioxidant activity (Oboh and Rocha 2007). Flavonoids, a type of phenolic, have been found have the function to anti-cancer and heart diseases (Filippos et al. 2007). Some triterpenoids exhibit antifungal, antibacterial, antiprotozoal, and anti-inflammatory properties (Amoussa et al. 2016; Vil et al. 2019).

Triterpenoids, phenolics, and flavonoids are the principal components in the alcohol-soluble parts of sanghuang mushrooms and are also their main active ingredients (Chen et al. 2019). Up to now, the fruiting bodies of the *SS* strain have failed to be artificially cultivated. Furthermore, they are hard and not very palatable. Therefore, we aimed to use a solid state fermentation (SSF) method to obtain desirable compounds from SS. SSF techniques, such as growing fungi on cooked grain, have traditionally been used to produce nutraceuticals in Asia for centuries (Murooka and Yamshita 2008). Most of the antioxidants we consume have a plant origin (Ejelonu et al. 2013). Cereal grains are significant sources of dietary antioxidants due to the abundant antioxidant compounds in their bran and germ, such as phenolic acid, anthocyanin, proanthocyanidin, γ-oryzanol and alkylresorcinols (Dykes and Rooney 2007). Cereal grains are not only a staple human food consumed all over the world but can also provide a high-quality growth substrate for mushrooms. Used the SSF method with *S. sanghuang,* it is possible to obtain an SS-grain fermented mixture that can be eaten directly. It is palatable and contains active ingredients from both the grains and the secondary metabolites of the fungus.

The nutritional compositions of grains are quite different (Mozaffarian et al. 2013). Few reports have investigated how these differences can affect the active compound contents and antioxidant activities of grains fermented by *S. sanghuang*. Therefore, the aim of this study was to assess the nutrient profiles, functional compositions, and antioxidant activities of seven different grains fermented with SS, and investigate the relationships between antioxidant capacities and bioactive compound/nutritional composition profiles. We hope to provide a novel method of producing better nutraceuticals and functional foods.

## Material and methods

### Materials and chemicals

A strain of *S. sanghuang* was obtained from the China General Microbiological Culture Collection Center, Beijing, PR China, for use in the present study. It was cultivated and maintained in potato dextrose agar (PDA). The seven grains used to prepare SSF substrates (oats, barley, millet, rice, buckwheat, corn, and coix seed) were purchased from a local grocery store. The oats, barley, millet, buckwheat, and corn originated from Liaoning Province, the coix seed was from Peking, and the rice was from Heilongjiang Province, China.

### Mycelium solid-state fermentation

Mycelia of *S. sanghuang* were gently attached to 9 cm-diameter plates of PDA. After 15 days’ incubation at 25 °C and with a 12:12 h light/dark cycle, 5 mm-diameter discs with SS strain colonies were inoculated in 50 mL Erlenmeyer flasks with potato dextrose broth with growing mycelia and shaken at 100 rpm at 25 ± 1 °C for 13–15 days. The liquid-fermentation mycelia were then homogenized in a sterile blender. Each SSF container was inoculated with 3 mL of mycelial homogenate onto different grain substrates. Before being inoculated, the seven grains were soaked in hot water for 1 h with stirring after being completely washed. The SSF substrate was composed of different grains (130 ± 5 g wet weight) supplemented with 1% CaCO_3_ and a water content of up to 45% in tissue-culture containers. The fermentation grain substrates were autoclaved at 121 °C for 120 min and then cooled prior to inoculation. After the fungal mycelia overgrew the grain substrates at 25 °C for several days, the hermetically sealed containers were opened to reduce humidity and the fungi allowed to continue growing for 7 days. The fermented mixtures were dried for 48 h at 60 °C, then milled with a food grinder and sieved through a 0.8-mm sieve for further analysis.

### Proximate composition

The AOAC (2005) methods were used to measure the proximate composition of the fungal substance products. The crude protein (N × 4.38), crude fat, starch, and ash contents were estimated by the Kjeldahl method (978.04), a Soxhlet apparatus (920.39), the enzymatic digestion method (2002.02) and incineration at 485 °C (920.05), respectively. Total carbohydrates were calculated using the formula: Total carbohydrates = (dry weight) − (crude protein weight) − (fat weight) − (mineral salts weight). The content of soluble protein was measured using a BCA protein assay kit (Shanghai Labaide Biotechnology Co., Ltd., PRB-000500/PRB250) following the method of Smith et al (1985). The soluble sugars in the samples were detected simultaneously using a phenol–sulfuric acid method (Hitachi Merck, Tokyo, Japan). All experiments were repeated in triplicate.

### Extraction and analysis of bioactive compounds

The preparation of extracts was adapted from the methods described by Kowalski (2009) with slight modifications. A total of 1 g of the powdered fungal substances was stirred with 25 mL of 70% methanol (or a 4:6 acetone-hexane mixture for carotenoids) into conical flasks. The samples were then extracted with an ultrasonic generator for 0.5 h, centrifuged and the obtained extracts were filtered by filter paper. The insoluble residue was extracted again as described above. The supernatant was collected and adjusted to 50 mL and properly protected and stored at 4 °C for further analysis, except for the assays of total triterpenoids.

#### Determination of carotenoids (CAs)

The carotenoid contents were analyzed according to the method described by Barros et al (2007) with the following modifications. The absorbance of the supernatant was measured as spectrophotometer at 453 nm and 663 nm. The concentration of carotenoids was calculated according to the formula:

$${\text{Carotenoids}}\left( {{{\upmu} g}/{\text{g powder}}} \right)\, = \,\left( 0.{\text{216A}}_{{{663}}} \, + \,0.{\text{452A}}_{{{453}}}  \right)\, \times \,V\, \times \,N/W$$

where *V* is the amount of solvent (mL), *N* is the dilution fold, and *W* is the sample weight (g).

#### Determination of total soluble phenolics (TSP)

The total soluble phenolic content was determined by the Folin–Ciocalteu method (Vermerris and Nicholson 2006). The total phenolic content was expressed as mg gallic acid equivalent per 100 g of dry sample (mg GAE/100 g dry wt).

#### Determination of total insoluble phenolics (TIP)

Due to the grain containing a large number of insoluble phenolics, and because we aimed to understand the sources of soluble phenolics in the fermentation of SS, we determined the insoluble phenolic contents of the grain substrates and SS-grain fermentation mixture. The insoluble phenolic fraction was extracted from the extract residue according to the methods described by Zhang et al (2010).

#### Determination of total flavonoids (TF)

Total flavonoid content was determined according to the method of Guo et al (2011). Results are expressed as catechin equivalent per gram of dry extract (mg QE/100 g dry wt).

#### Determination of total triterpenoids (TTP)

The total triterpenoid contents were determined according to the methods of Zhou et al (2011). The total triterpenoid content was expressed as the percentage of ursolic acid equivalent per gram of dry extract.

### Determination of antioxidant activity

We chose four complementary assays that were used for the analysis of antioxidant activity, which are include radical scavenging assay (ABTS^+^), DPPH free radical scavenging capacity (DPPH), total antioxidant activity assay (T-AOC), and DNA damage protecting activity(DNAp). The extracts were prepared as described in “Extraction and analysis of bioactive compounds” section.

#### Total antioxidant activity assay (T-AOC)

Total antioxidant capacity was conducted using the T-AOC assay (Jiancheng Nanjing), which utilizes the conversion of Fe^3+^ ions to Fe^2+^ through endogenous protein and small molecule antioxidants and monitors the reactions of Fe^2+^ ions chelated with a colorimetric probe, which form an orange color with an absorbance peak at 520 nm. The T-AOC was expressed as U/mg SS-grain fermentation power, where 1 U means 1 mg of SS-grain powder increases the value of absorption OD by 0.01 points per minute at 37 °C.

#### Determination of DPPH free radical scavenging capacity (DPPH)

DPPH was determined using the method described by Oboh and Rocha (2007). The absorbance was measured at 517 nm by the spectrophotometer and the extraction solvent was used as a control. The percentage inhibition of the radicals represents the DPPH due to the antioxidant activity of SS-grain extracts, which was calculated using the following formula of inhibition:

$$\% = \left( {\left( {{\text{A}}_{{{\text{control}}}} - {\text{ A}}_{{{\text{sample}}}} } \right)/{\text{A}}_{{{\text{control}}}} } \right) \, \times \, 100\%$$

#### Determination of radical scavenging assay (ABTS^+^)

A colorimetric method was used for measured ABTS^+^ (Oboh and Rocha 2007). The supernatant was read read at 734 nm after 30 s reaction in a spectrophotometer set at 30 °C. The ABTS was expressed as the percentage inhibition of antioxidant activity of the SS-grain extracts and was calculated using the following formula of inhibition:

$$\% = \left( {({\text{A}}_{{{\text{ABTS}}}} ^{ + } \pm {\text{ A}}_{{{\text{sample}}}} )/{\text{A}}_{{{\text{ABTS}}}} ^{ + } } \right)\; \times \;100\%$$

where A_ABTS_^+^ and A_sample_ are the absorbance radical cations without antioxidant and with sample extract, respectively.

#### Site-specific hydroxyl-radical-mediated DNA damage-protecting activity (DNAp)

DNA strand breaks were measured by the procedure described by Yeung et al (2002) with some modifications. Briefly, 1 μL plasmid pUC19 DNA (0.5 g) was incubated with 1 μL of 1 mM FeSO_4_, 1 μL of 10% H_2_O_2_, and 3 μL of ethanol extracts. The final volume was made up to 15 μL with 0.05 M phosphate buffer buffer (pH 7.0). The mixture was incubated in a water bath for 30 min at 37 °C. The negative and positive controls consisted of 3 μL of H_2_O and rutin instead of sample, respectively. After incubation, the sample was immediately electrophoresed in a 1.5% agarose gel along with 3 µL ethidium bromide. The plasmids generally have three states: super coiled circular DNA (ScDNA), an open circular form (OcDNA), and linear DNA (lnDNA). These correspond to three bands observed in agarose gel electrophoresis. The DNA protection ability was quantified by the ratio of the three DNA bands. Image J software was used to convert the brightness of the DNA bands into grayscale data. Then, the DNA protection ability was calculated using the formula:

$${\text{Inhibition}}\% \, = \, \left( {1 - \left( {{\text{G}}_{{{\text{OcDNA}}}} + \, 1/2{\text{G}}_{{{\text{InDNA}}}} } \right)/\left( {{\text{G}}_{{{\text{ScDNA}}}} + {\text{ G}}_{{{\text{OcDNA}}}} + {\text{ G}}_{{{\text{InDNA}}}} } \right)} \right) \, \times \, 100$$

where G_ScDNA_, G_OcDNA,_ and G_InDNA_ are the grayscale parameters of ScDNA, OcDNA, and InDNA, respectively.

### Statistical analysis

All data are averages of three independent assays and are expressed as means ± standard deviation (SD). Differences between groups were tested by two-way ANOVA. Principal component analysis (PCA) was used to arrange a set of cases into clusters. Cluster analysis was performed using hierarchical cluster analysis based on the Ward variance method. In addition, changes in bioactive compound contents and antioxidant activity due to SS fermentation were corrected with corresponding grain controls and remarked as *Δ*CA/TP/TF/TTP and *Δ*T-AOC/ABTS^+^/DPPH/DNAp respectively. The variable *Δ*T-AOC/ABTS^+^/DPPH/*Δ*DNAp was then separately correlated to the variables *Δ*CA, *Δ*TP, *Δ*TF and *Δ*TTP using the method of stepwise multivariate correlation and a linear correlation analysis was also performed between paired variables of corrected antioxidant activity in DPS software. Differences were considered significant when *P* < 0.05 (Tang and Feng 2007).

## Results and discussion

### Proximate composition of seven SS-grain fermentation

The results for seven macronutrients in the studied SS-grain samples and their controls are shown in Table 1. Carbohydrates were the most abundant macronutrients, while fat and ash contents were lower. In the SS-grain mixture, the carbohydrate contents were 75.03–89.83%, which is similar to that of unfermented grain (76.06–89.00%). The crude fat contents were 0.57–7.71% in the seven different SS-grain mixtures. After fermentation by SS, the fat contents decreased significantly (*F*_1,41_ = 47.6, *P* < 0.01). Starches ranged from 18.06–53.57%, decreasing to 5–56% after fermentation. There were four nutrient types that increased significantly after SS mycelium fermentation according to a fixed-effects model: crude protein (*F*_1,41_ = 111.1, *P* < 0.01), soluble protein (*F*_1,41_ = 595.7, *P* < 0.01), soluble sugar (*F*_1,41_ = 51.4, *P* < 0.01) and ash (*F*_1,41_ = 227.3, *P* < 0.01). The SS consumed fat and starch in the grains and turned them into soluble protein and soluble sugar. These results reveal that the fermentation of seven different grains by SS species not only produced many bioactive compounds but also changed the nutrients in the grains.

**Table 1:** Macronutrients of SS-grain fermention mixture and the grain substrate control in dried powder

Sample item	Crude protein^a^ (g/100 g powder)	Crude fat^a^ (%)	Carbohydrate^a^ (%)	Starch^a^ (%)	Soluble protein^a^ (mg/g powder)	Soluble sugar^a^ (g/100 g powder)	Total ash^a^ (g/100 g powder)
Oat	11.93 ± 0.35c	8.57 ± 0.20a	77.73 ± 0.69de	32.05 ± 0.53d	2.09 ± 0.17d	14.42 ± 0.78de	1.77 ± 0.17cdef
Barley	10.41 ± 0.72cd	3.10 ± 0.49cd	85.27 ± 0.15c	18.06 ± 0.30ef	1.36 ± 0.10d	27.67 ± 0.80cd	1.23 ± 0.19fgh
Millet	9.21 ± 0.51d	2.93 ± 0.18cd	86.81 ± 0.37bc	48.71 ± 0.13ab	1.38 ± 0.08d	9.30 ± 1.88e	1.03 ± 0.03ghi
Rice	9.26 ± 0.89d	1.27 ± 0.09f	89.00 ± 0.52ab	53.57 ± 0.57a	1.41 ± 0.09d	11.15 ± 1.70e	0.43 ± 0.12ig
Buckwheat	15.28 ± 0.64b	3.80 ± 0.21c	78.73 ± 0.73d	51.06 ± 0.75a	3.45 ± 1.35cd	23.66 ± 2.18cde	2.167 ± 0.18cd
Corn	8.62 ± 0.66d	1.63 ± 0.19ef	89.48 ± 0.45a	21.56 ± 0.46e	1.27 ± 0.03d	15.01 ± 1.49de	0.267 ± 0.03j
Coix seed	15.02 ± 0.42b	6.97 ± 0.19b	76.06 ± 0.53ef	32.21 ± 0.27d	2.02 ± 0.26d	20.82 ± 0.80cde	1.93 ± 0.15cde
SS-Oat	15.41 ± 0.83b	6.56 ± 0.34b	75.03 ± 0.81fg	11.45 ± 0.46g	8.56 ± 0.52b	13.91 ± 2.52de	2.93 ± 0.03ab
SS-Barley	18.83 ± 0.57a	2.53 ± 0.30de	75.47 ± 0.15ef	14.87 ± 0.95fg	12.25 ± 1.01a	33.48 ± 1.38bc	3.17 ± 0.12a
SS-Millet	8.60 ± 0.57d	0.57 ± 0.23f	89.83 ± 0.13a	42.17 ± 0.99c	6.37 ± 0.36bc	22.27 ± 1.34cde	1.00 ± 0.15hi
SS-Rice	12.41 ± 0.60c	1.17 ± 0.09f	84.77 ± 0.18c	31.67 ± 0.15d	14.80 ± 0.98a	26.78 ± 8.4cde	1.67 ± 0.09defg
SS-Buckwheat	15.33 ± 0.55b	3.43 ± 0.04cd	78.83 ± 0.38d	22.47 ± 1.38e	4.54 ± 0.75cd	12.36 ± 0.97de	2.40 ± 0.06bc
SS-Corn	8.42 ± 0.43d	0.90 ± 0.15f	89.25 ± 0.77ab	20.57 ± 0.40e	14.66 ± 0.57a	45.62 ± 0.59ab	1.47 ± 0.09efgh
SS-Coixseed	16.89 ± 0.20ab	7.17 ± 0.12b	72.76 ± 0.21g	45.59 ± 0.80bc	12.57 ± 0.99a	55.65 ± 9.00a	3.20 ± 0.15a
*F*_13,41_ value^b^	75.62**	132.84**	161.37**	208.68**	63.74**	78.23**	301.23**

^a^Data were expressed as mean ± standard deviation (n = 3). The data in the same column marked with different small case letters are significantly different (Tukey’s HSD, *P* < 0.05).^b^ *F* value in two-way ANOVA: *P* < 0.05 (*) or 0.01 (**)

The fungal mycelia fully overgrew the grain substrates in an average of 12.0 d in oats, 10.5 d in barley, 16.0 d in millet, 25.0 d in rice, 17.5 d in buckwheat, 8 d in corn, and 16.8 d in coix seed. The growth rates of SS differed significantly by grain type (*F*_6,27_ = 64.8, *P* < 0.01). Soluble protein can used to indirectly determine the fungal biomass present during SSF (Steudler and Bley 2015). According to changes in soluble protein, we found that rice (13.39 mg/g powder) and corn (13.39 mg/g powder) produced the most fungal biomass, while buckwheat produced the least (1.09 mg/g powder). There were no relationships between mycelial growth rate and biomass (*F*_1,6_ = 0.015, *P* = 0.9).

Moreover, the correlation coefficients show that there was a significant positive correlation between mycelial growth rate and grain starch content (*r* = 0.85, *P* < 0.01). As a kind of wood-decaying fungi, the mycelia of SS have many starch-degrading enzymes. These seem to be involved in the initial degradation of starch, which provides nutrients and allows colonization into the wood (Tanaka et al. 2020). Starch is a kind of polysaccharide with two different structures (amylose and amylopectin). Amylose is a linear α-1,4-linked glucan, and amylopectin has branches of short α-1,4-linked glucan with a α-1,6-linkage. Corn, oats, and barley are high-amylopectin grains, and this research indicates that SS degrades amylopectin more easily than amylose.

Ash usually indicates the total mineral content in a food sample (Ulziijargal and Mau 2011). In Table 1, the ash contents of all SS-grains were higher than that of the grain culture. The SS-grains had low-fat contents and high contents of soluble proteins and minerals, which are beneficial to human health.

### Bioactive compound content of seven SS-grain fermentation

**Fig. 1:**
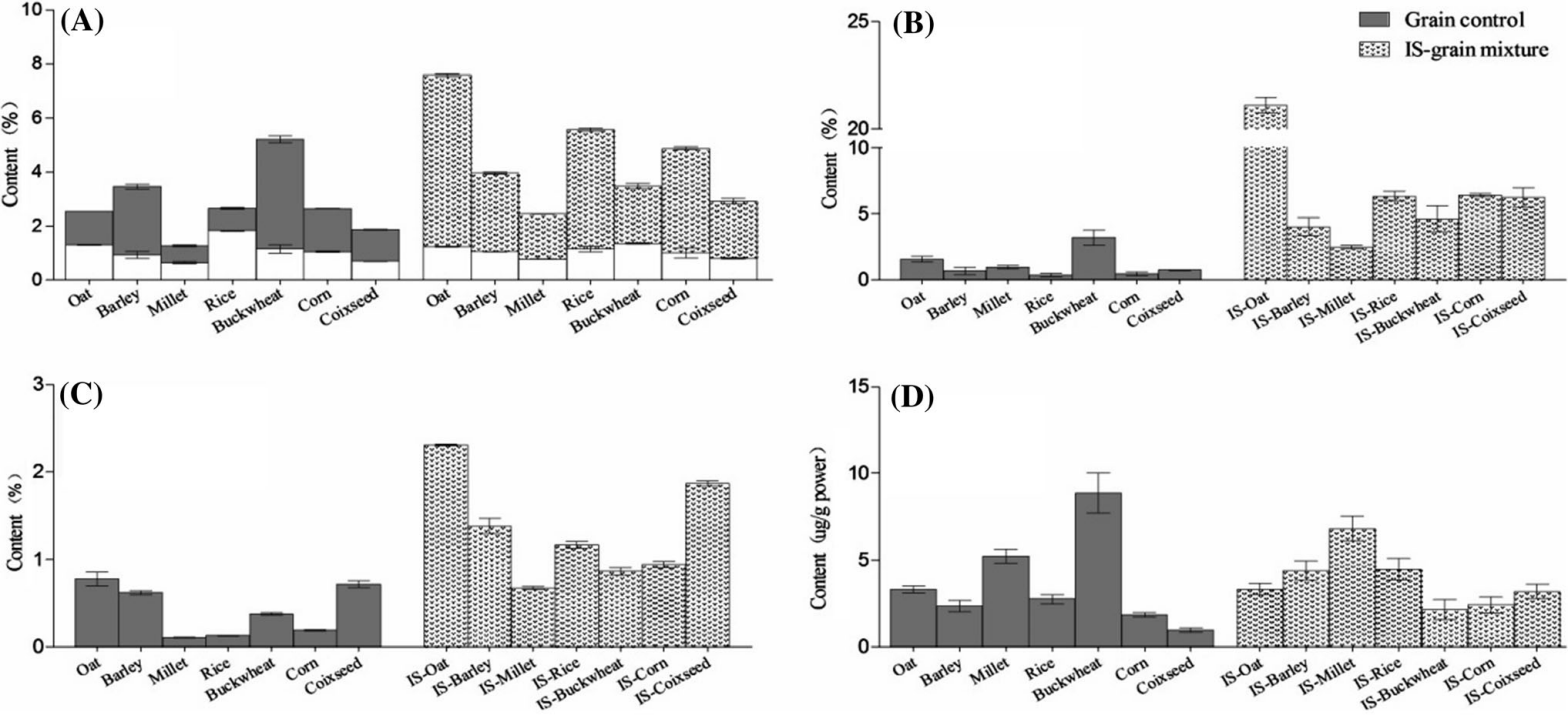
Four bioactive compounds content of seven SS-grain fermentation and compared with corresponding grain-only powder. **a**Total contents of phenolic acids, which including soluble and insoluble phenol. The blank column (□) in figure A represents the insoluble phenol in corresponding samples. **b** Total contents of flavonoids. **c** Total contents of triterpenoids. **d** Total contents of carotinoid

The total phenolic contents of the extracts from the fermented and unfermented grains are shown in Fig. 1a. The results indicate that SS fermentation distinctly changed the TSP (*F*_1,41_ = 2319.2, *P* < 0.01) but did not change the TIP (*P* = 0.32), which is contained in the untreated grain. Our data indicate that SS metabolism in grain can produce polyphenols, but does not convert the TIP in the grain into TSP. The TSP contents varied from 1.66 to 6.35 (mg GAE/100 g dry wt) in SS-grain fermentation and from 0.62 to 4.06 (mg GAE/100 g dry wt) in the controls. The data show that the amounts of phenolics in all seven fermentation grains differed significantly. By comparing the compositions of the 7 SS-grain mixtures, it was determined that the highest phenolic content was in the SS-oat fermentation mixture. Except for SS-buckwheat mixture, the total phenolics in all six other SS-grain mixtures increased compared to corresponding controls. However, the TSP content in the SS-buckwheat mixture decreased by 48% after fermentation.

The TF contents of the tested samples are shown in Fig. 1b. There were significant differences (*F*_13,41_ = 425.8, *P* < 0.01) among the samples and the TF contents were effectively improved by SS fermentation (*F*_1,41_ = 2184.0, *P* < 0.01). The highest TF content was obtained from the SS-oat mixture (21.09 mg rutin/g dry wt), while the SS-rice mixture produced the lowest flavonoid content of the fermented samples. The flavonoids have a wide range of biological activities and pharmacological and medicinal properties; therefore, there is much interest in the metabolism of these compounds (Moghaddam and Mehdizadeh 2015). In order to obtain their potential benefits, obtaining high quantities of TF via fermentation is important. Accordingly, oats were considered the best substrate for special applications as the SS-oat mixtures exhibited high amounts of flavonoids after SS fermentation.

The TTP contents in the seven fermentation samples were significantly higher than those of the corresponding grains (*F*_1,41_ = 1706.5, *p* < 0.01). The highest TTP content was in the SS-oat mixture (2.31%), which is approximately threefold higher than that of unfermented oats. The TTP contents of the SS-coix seed mixture reached 1.87%, which is much more than those of the other SS-grain mixtures (Fig. 1c). Figure 1d presents the carotenoid contents of SS-grain and grain-only samples. Similar to the TSP content results, the carotenoid content of SS-buckwheat was 414% lower than that of the buckwheat control. In general, there were no differences between the SS-grain and grain samples (*P* = 0.46).

### Antioxidant activity of the seven fermented SS-grain mixtures

**Fig. 2:**
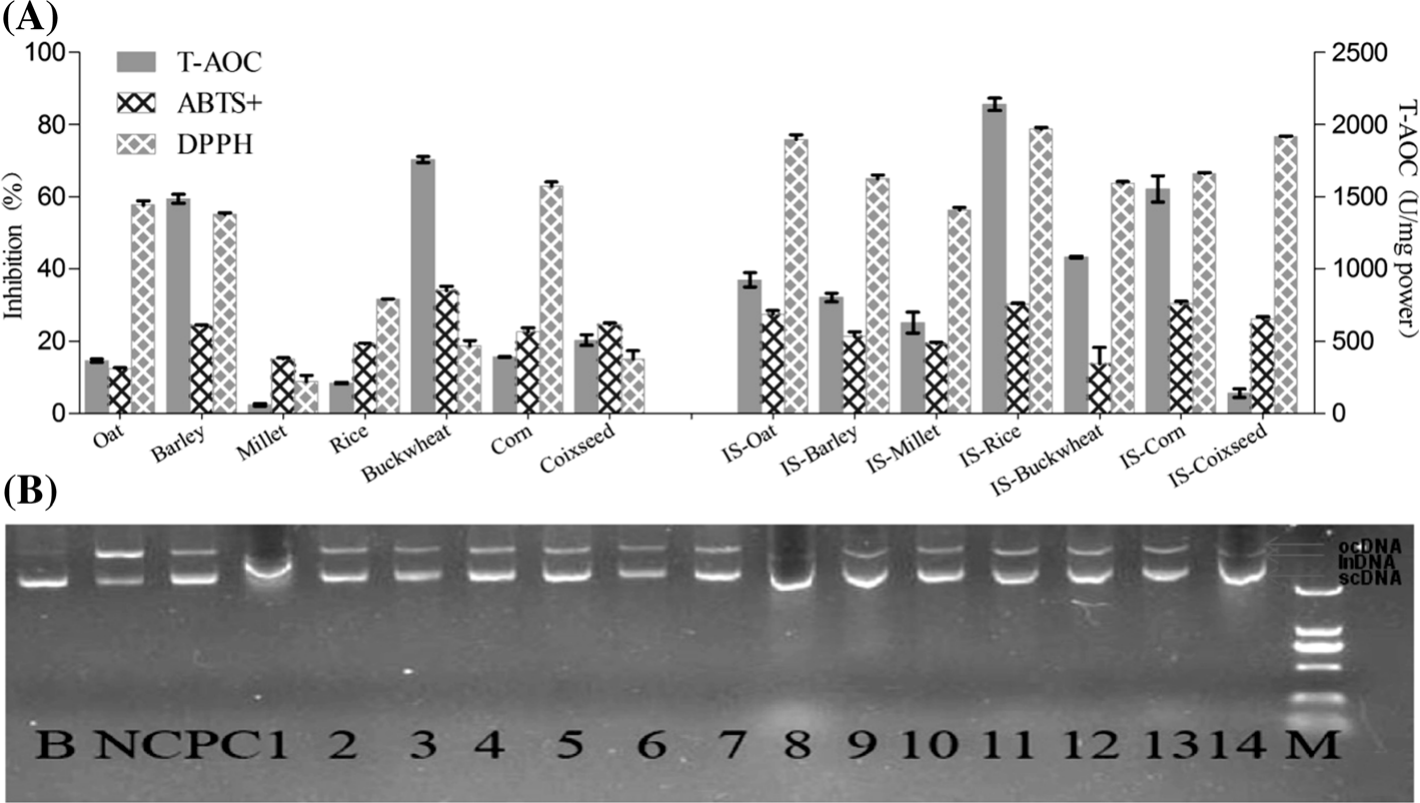
Antioxidant activity of 70% ethanol extract of SS-grain mixture and corresponding control grain. **a** Antioxidant capacities of T-AOC, DPPH, and ABTS. **b** The protection of supercoiled DNA (plasmid pUC19) with samples. Lane B is untreated DNA (Blank control). Lane NC, FeSO_4_ + H_2_O_2_ treatedwith H_2_O (negative control). Lane PC, FeSO_4_ + H_2_O_2_ treated with rutin (positive control). Line M is a marker. Lane1-7, FeSO_4_ + H_2_O_2_ treated with SS-grain fermentation mixture extract. Lane 8–14, FeSO_4_ + H2O2 treated with grain extract. The number 1–7 represent Oat, Barley, Millet, Rice, Buckwheat, Corn and Coixseed grain-only respectively. The number 8–14 represent SS-Oat, SS-Barley, SS-Millet, SS-Rice, SS-Buckwheat, SS-Corn, and SS-Coixseed fermentation extract respectively

The T-AOC (expressed in U/g), ABTS radical scavenging activity, and DPPH free radical scavenging activity of the 14 samples (seven control, seven fermented) are presented in Fig. 2a. Relatively high T-TOC levels were recorded in SS-rice and SS-corn mixtures (2140.00 and 1555.56 U/g), while the SS-coix seed mixture showed the lowest antioxidant capacity (142.22 U/g). The T-AOC contents of the SS-barley, SS-buckwheat, and SS-coix seed mixtures were decreased significantly by fermentation with SS, while the other four fermented samples had higher levels than their non-fermented controls. The ABTS^+^ radical scavenging activity increased significantly after SS fermentation (*F*_1,41_ = 11.6, *P* = 0.002; Fig. 2a). All fermented samples exhibited higher ABTS radical scavenging activity than their controls, with the lowest value among the fermented samples apparent in the SS-oat mixture (12.38%). The DPPH free radical scavenging activity increased significantly after SS fermentation (*F*_1,41_ = 2175.3, *P* < 0.01; Fig. 2a). A relatively high DPPH scavenging ability was recorded, with inhibition of 75.82%, 78.74%, and 76.66% in SS-oat, SS-rise, and SS-coix seed mixtures, respectively, while the lowest DPPH scavenging ability was found in SS-millet (56.28%).

The protective effects of the ethanol extract obtained from the SS-grain mixtures on the damage induced by the combination of H_2_O_2_ (2.5 mmol/L) and UV radiation were studied in a pUC19 plasmid. Figure 2b shows that there are three types of DNA electrophoretic pattern after UV and H_2_O_2_ treatment in the absence or presence of samples or rutin. In blank contronl(lane B) of the agarose gel, the pUC19 plasmid showed a band corresponding to the native form of super-coiled circular DNA (Sc DNA). UV irradiation of the DNA with H_2_O_2_ resulted in the cleavage of Sc DNA to an open circular form (Oc DNA, lane NC). The positive control(lanes PC) revealed that the addition of rutin suppressed the formation of Oc DNA, indicating that the antioxidant compound offered protection to the native Sc DNA (Zhang et al. 2015). Lines 1–7 represent the DNA protection offered by the cereal grain ethanol extract, while lines 8–14 show it for the SS-grain mixture. Except for the oat sample, which resulted in the Sc DNA changing to Oc DNA, the other 13 samples all exhibited similar protective functions with Sc DNA. The DNA protection ability was quantified by ImageJ software (data not shown) but the different was not significant between grain controls and SS-fementation samples.

Antioxidants play an important role in preventing the formation of free radicals and other potentially toxic oxidizing species. There are three categories of antioxidant species that can be detected by a T-AOC kit: organic molecules (ascorbate, uric acid, GSH, vitamins, etc.), enzyme systems (GSH reductase, catalase, peroxidase, etc.), and proteins (albumin, transferrin, etc.). The T-AOC activity of the grains changed significantly with SS-fermentation (*F*_1,28_ = 276.81, *P* < 0.01). Those for barley, buckwheat, and coix seed declined, while those of the others increased 3–tenfold after SS fermentation. In this study, we also used water to extract the SS-grain fermentation powder. The data showed that the water extract had little antioxidant activity (data not shown). All the results above suggest that SS fermentation produces a large number of organic molecules but not antioxidants, enzymes, or proteins. DPPH is a stable radical at room temperature and can accept electron or hydrogen radicals to become a stable diamagnetic molecule. It has been used to determine the antioxidant activity of various neutral products (Hu and Kitts 2000; Soares et al. 1997). Due to hydrogen donating of the antioxidants, the free radicals of DPPH was thought to be removed (Liu et al. 2002). The DPPH scavenging activity of the SS-fermented extract was recorded as 56.28–78.74%, while that of the grain controls was 8.91–62.96%. The average DPPH in the SS-fermentation samples was 68.96%, which is 193% higher than that of the corresponding grains (*F*_1,28_ = 2175.29, *P* < 0.01). The ABTS^+^ radical scavenging activity is an indication of the potential of antioxidants to donate electrons or hydrogen atoms to deactivate these radical species (Bhuvaneswari et al. 2012). The reduced ABTS^+^ radical is colorless in a color-quenching reaction. The results show that all 14 samples could reduce oxidative damage caused by organic radicals. There were significant differences in the ABTS^+^ radical activities of the grain control extracts and the SS-grain fermentation samples (*F*_1,28_ = 11.65, *P* = 0.02). Except for buckwheat, the ABTS^+^ radical scavenging activity slightly increased after SS fermentation. The data show that the effects of SS fermentation on the antioxidant activities of T-AOC, DPPH, and ABTS^+^ were significant (*P* < 0.01). This finding suggests that the choice of fermentation substrate is as important as the SS strain itself in obtaining products with strong antiradical potential.

### Correlations

The coefficients of the correlations between the changes of bioactive compounds (*Δ*TSP, *Δ*TF, *Δ*TTP, and *Δ*CA) in SS fermented and initial nutrient and bioactive component in corresponding grain substrates are shown in Table 2. There were significant negative correlations between the changes in *Δ*TSP and *Δ*CA caused by SS fermentation and the contents of TSP in grain substrates, and between *Δ*CA and the contents of TF, CA and soluble protein in grain substrates. This indicates that the bioactive compounds in the grains may cause negative feedback to the enzymes in the secondary metabolite synthesis pathway in SS. In addition, there were significant positive correlations between *Δ*TF and crude fat content. This correlation provides an important standard for development of an SSF formula for the SS strain.

**Table 2:** Coefficients of correlation (*r*) between changes in bioactive compounds (*Δ*TSP, *Δ*TF, *Δ*TTP, and *Δ*CA) in *S. sanghuang* fermented and proximate compositions and bioactive compounds of their corresponding grain substrates

	TSP^a^	TF^a^	TTP^a^	CA^a^	Crude protein^a^	Crude fat^a^	Carbohydrate^a^	Starch^a^	Soluble protein^a^	Soluble sugar^a^	Total ash^a^
*Δ*TSP	− 0.72*	− 0.33	0.11	− 0.55	− 0.47	0.25	0.16	− 0.12	− 0.5	− 0.61	− 0.4
*Δ*TF	− 0.22	0.28	0.57	− 0.22	0.11	0.71*	− 0.43	− 0.2	0.1	− 0.19	0.22
*Δ*TTP	− 0.47	− 0.07	0.62	− 0.55	0.11	0.7	− 0.41	− 0.21	− 0.12	− 0.19	0.14
*Δ*CA	− 0.82*	− 0.83**	0.05	− 0.86**	− 0.55	− 0.03	0.35	− 0.38	− 0.90**	− 0.3	− 0.5

^a^Correlation was analyzed by Pearson product-moment correlation coefficient

Correlation is significant at *P* < 0.05 (*) or 0.01 (**)

**Fig. 3:**
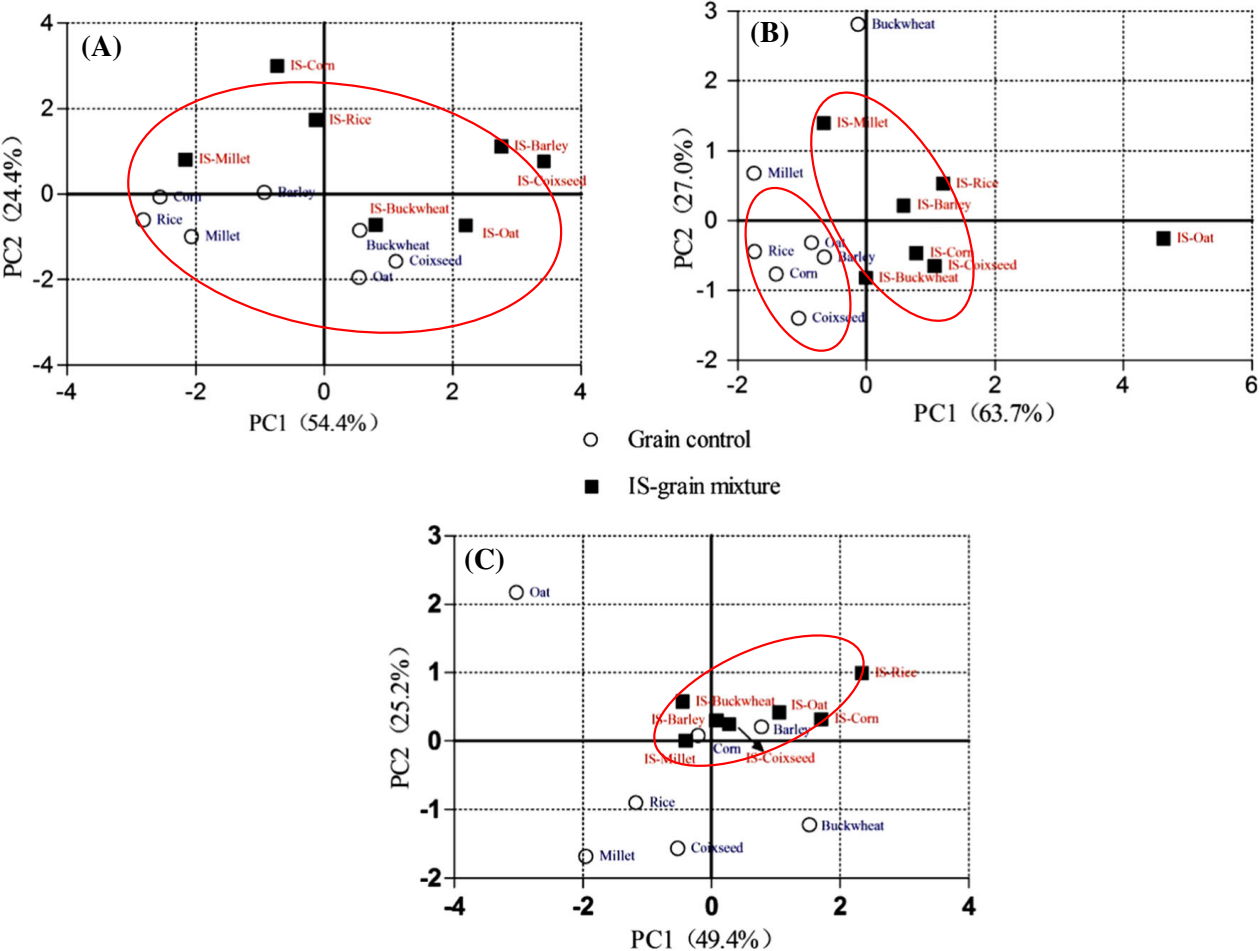
Principal component analysis of 14samples (7 SS-grain Fermentation products and 7 corresponding grain control). (A) PCA based on its seven of proximate composition. **b** PCA based on four of bioactive compounds (CA, TSP, TF, and TTP). **c** PCA based on T-AOC, DPPH, ABTS^+^, and DNAp

Differences and similarities between the seven SS-grain fermentation mixtures and their corresponding controls in terms of seven proximate compositions, four bioactive compounds, and four antioxidant activity indexes were visualized by PCA analysis (Figs. 3a–c). Fermentation changed the nutrient structure, but all 14 samples showed similarities in proximate composition when considered as a complete data set (Fig. 3a). The SS-grain mixtures were significantly different from the unfermentated grains only in these properties of the four bioactive compounds. The first two principal components (Fig. 3b) cumulatively explained 90.7% of the total variance. The TTP and TSP contents were the most important factors in the first and second principal components, respectively. There was one SS-grain (SS-oat) sample and one control grain powder(buckwheat) not be grouped, because their TTP contents were significantly higher than those of the corresponding group. After fermentation, the antioxidant activity of the SS-grain sample was changed distinguished compared to the control samples and similarities in one group (Fig. 3c).

**Fig. 4:**
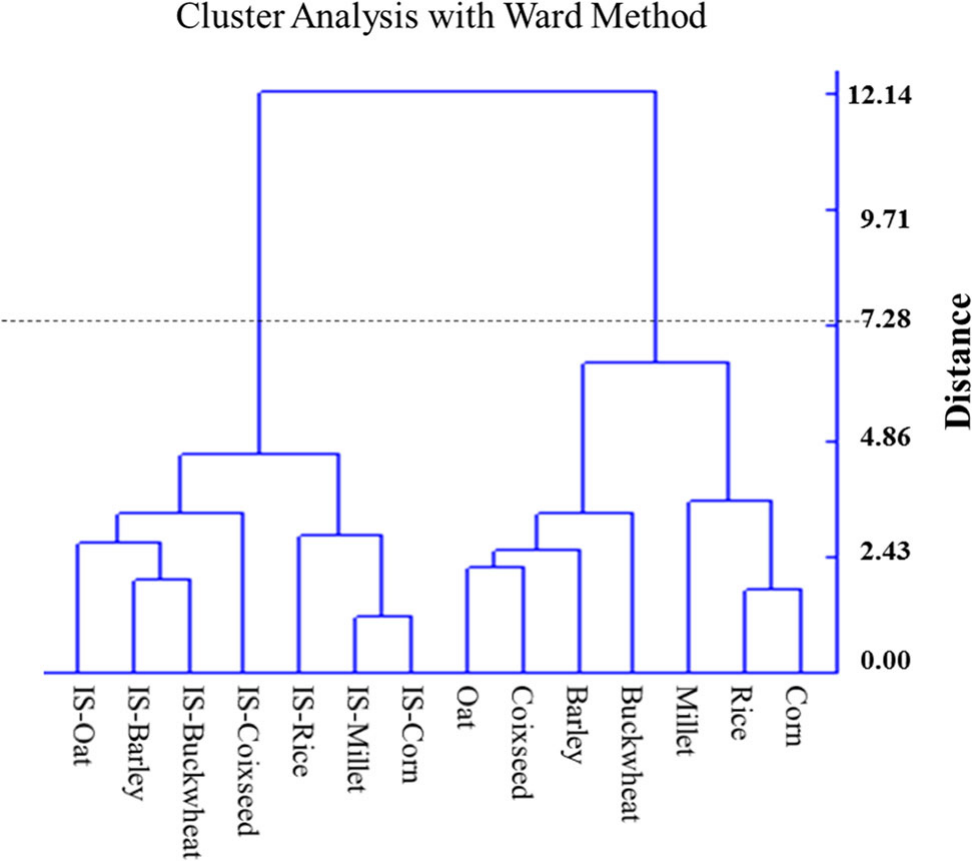
Grouping of 7 SS-grain fermentation mixture and corresponding substrate grain according to content and four of antioxidant activity using the Ward method

Cluster analysis was also used to identify differences among the 14 samples. According to seven proximate compositions, four bioactive compound contents, and four antioxidant activities (the dataset calculated after logarithm), the cluster analysis subdivided the 14 samples into two major groups (Fig. 4). Group I composed of fermented grains, while Group II consisted of all control grain samples. The results indicate that SS fermentation changed the physical and chemical characteristics of grains classified as a cluster.

Based on the corrected data caculated by Figs. 1 and 2, the *Δ*T-AOC, *Δ*ABTS, *Δ*DPPH, and *Δ*DNAp oxidation were significantly correlated to part of *Δ*TSP, *Δ*TF, *Δ*TTP, and *Δ*CA, yielding:

$$\begin{aligned} \varDelta {\text{T-AOC }} & = { 412}.{6 } + { 9}0{9}.{9}\varDelta {\text{TSP }} - { 227}.{4}\varDelta {\text{TF }} \\ & \quad - \;{221.{2}}\varDelta {\text{CA }}(r^{{2}} = 0.{97},F_{{{3},{3}}} = {36}.{9},P < \, {0.0{1}}); \\ \end{aligned}$$

$$\varDelta {\text{ABTS}}^{ + } = - {4}.{1} + {3}.{9}\varDelta {\text{TSP }} + { 1}.{2}0\varDelta {\text{CA }}(r^{{2}} = 0.{98},F_{{{2},{4}}} = {77}.{6},P < 0.0{1});$$

$$\begin{array}{lll} \varDelta {\text{DPPH }}  =  37.1 + 18.0\varDelta {\text{TSP}} - 39.1\varDelta {\text{TTP}} \\
\quad+ 3.0\varDelta {\text{CA}}\,(r^{2} = 0.98,F_{3,3} = 48.0,P < 0.01);&{\text{and}}\;\varDelta {\text{DNAp}}  = - \;9.8 + 2.9\varDelta {\text{TF}}\;(r^{2} = 0.95,F_{1,5} = 62.4,P < 0.01).\end{array}
$$

Correlation analysis indicated that the (1) *Δ*T-AOC were significently correlated to *Δ*TSP, *Δ*TF and *Δ*CA contents (0.973); (2) The *Δ*DPPH were significently correlated to *Δ*TSP, *Δ*TTP, and *Δ*CA contents(0.981); (3) The *Δ*ABTS^+^ were significently correlated to *Δ*TSP and *Δ*CA contents and (0.979); and (4) a significant positive correlation was found between *Δ*DNAp and *Δ*TF. Based on analysis of the fitted coefficients, the antioxidant activity mainly depended on the TSP (*P* < 0.05 in Student’s *t*-tests of their coefficients).The antioxidant potential activity of polyphenols comes from their ability to scavenge free radicals, donate hydrogen atoms or electrons, or chelate metal cations. These all depend on the presence of electron-withdrawing/donating substituents in their structure and the number and arrangement of hydroxyl groups (Zhang et al. 2003). Cai et al. (2004) found that phenolic compounds, which include flavonoids, phenolic acids, and condensed tannins, are the main contributors to the antioxidant capacities of plants. Our results show that total phenolic compounds are major factors in antioxidant anilities of SS-fermentation extracts, which is consistent with studies on plants. Similar to our results, Bajalana et al (2016) demonstrated that different phenolic contents are major factors deciding the antioxidant capacity of *lavandin* industrial extracts. Other researchers (Corral-Aguayo et al. 2008; Gunaratne et al. 2013) have found a linear relationship between total phenolic compounds and antioxidant activity. In our study, antioxidant activity was related to the concentrations of TSP, TF, and CA, but the relationship was not linear. Fermentation metabolites are complex and the use of a 70% methanol solvent can extract part of the water-soluble substances separately from the alcohol-soluble substances. The antioxidant action may be increased by other substances such as polysaccharides, tocopherols, vitamins, and so on (Cheung et al. 2003; Zhang et al. 2013). We need further research to find out what kinds of materials play key roles in such reactions.

In addition, this research indicates that flavonoids, a kind of polyphenols, had a negative correlation with T-AOC, while there was a positive correlation between TF and DNAp (0.955) at a 1% probability level. TTP has a negative correlation with changes in DPPH, which means that triterpenoids have bioactivity but no antioxidant activity. Furthermore, a significant positive correlation was found between *Δ*T-AOC and *Δ*DPPH (*r*^2^ = 0.67, *F*_1,14_ = 17.7, *P* = 0.028) and *Δ*DPPH and *Δ*ABTS (*r*^2^ = 0.94, *F*_1,14_ = 10.9, *P* = 0.0018), respectively, in linear correlation analysis, but this was absent between *Δ*DNAp with the other three. These suggest some independence of DNA protection activity.

## Conclusion

This study demonstrates that grain can produce more bioactive compounds and antioxidants when fermented with SS. Antioxidant assays are reliable but there were no significant correlations between the T-AOC, DPPH, ABTS, and DNAp values. There was a high correlation between antioxidant capacity and bioactive compound levels after fermentation, indicating that *SS* can produce a large number of bioactive substances by SSF. SS-oats displayed the highest total bioactive compound contents and antioxidant capacities among the fermented samples and could be used to develop health products for the prevention of disease caused by oxidative stress.
